# Gentamicin Arrests Cancer Cell Growth: The Intriguing Involvement of Nuclear Sphingomyelin Metabolism

**DOI:** 10.3390/ijms16022307

**Published:** 2015-01-22

**Authors:** Michela Codini, Samuela Cataldi, Francesco Saverio Ambesi-Impiombato, Andrea Lazzarini, Alessandro Floridi, Remo Lazzarini, Francesco Curcio, Tommaso Beccari, Elisabetta Albi

**Affiliations:** 1 Department of Pharmaceutical Science, University of Perugia, Perugia 06122, Italy; E-Mails: codini@virgilio.it (M.C.); samuelacataldi@libero.it (S.C.); tommaso.beccari@unipg.it (T.B.); 2 Department of Clinical and Biological Sciences, University of Udine, Udine 33100, Italy; E-Mails: ambesis@me.com (F.S.A.-I.); curcio@uniud.it (F.C.); 3 Laboratory of Nuclear Lipid BioPathology, CRABiON, Perugia 06122, Italy; E-Mails: andrylazza@gmail.it (A.L.); info@crabion.it (A.F.); remo30@libero.it (R.L.)

**Keywords:** gentamicin, lymphoma, nucleus, sphingomyelinase, sphingomyelin synthase

## Abstract

The use of gentamicin for the treatment of bacterial infection has always been an interesting and highly speculated issue for the scientific community. Conversely, its effect on cancer cells has been very little investigated. We studied the effect of high doses of gentamicin on non-Hodgkin’s T-cell human lymphoblastic lymphoma (SUP-T1). We showed that gentamicin delayed cell growth and induced cell death in lymphoma cells with a rather mild effect on lymphocytes. In SUP-T1 cells, GAPDH, B2M, CDKN1A and CDKN1B were down-expressed in comparison with lymphocytes. Gentamicin treatment in SUP-T1 cells restored the expression of GAPDH, B2M and CDKN1A to values similar to those of lymphocytes and caused overexpression of CDKN1B. The drug acted via sphingomyelin metabolism; in whole cells, sphingomyelinase activity was stimulated, whereas in pur**i**fied nuclei, sphingomyelinase activity was inhibited and that of sphingomyelin-synthase was stimulated, with a consequent high level of nuclear sphingomyelin content. We suggest that the increase of nuclear sphingomyelin might enrich the nucleus of lipid microdomains that act as a platform for active chromatin and, thus, might be responsible for gene expression. It is possible that in lymphoblastic lymphoma, high doses of gentamicin induce a beneficial therapeutic outcome.

## 1. Introduction

Gentamicin (GM) is an aminoglycoside antibiotic widely used for the treatment of bacterial infection. While the bactericidal effects of aminoglycosides are due to binding to the 30S subunit of the bacterial ribosome, aminoglycosides could affect protein synthesis, intracellular calcium levels and the levels of reactive oxygen species (ROS) in eukaryotic cells [1]. At therapeutic concentrations, GM accumulated in lysosomes and stimulated apoptosis in kidney proximal tubular cells by ROS production [2] and increasing the expression of heat shock protein (HSP) 72 [3], induced an oxidative stress status by increasing free radical formation and lipid peroxidation in the testis [4] and increased mitochondrial p53 activity in promoting hair cell death [5]. It has been recently demonstrated that GM induced cell death via the ceramide/sphingomyelin (SM) pathway [6].

The involvement of SM metabolism in blood malignance, as well as lipid rafts rich in SM and cholesterol (CHO) as targets of the therapeutic treatment of lymphoma [7] have been described. Bezombes *et al.* [8] demonstrated that rituximab-induced growth inhibition of B-lymphoma cells was mediated through a ceramide-triggered signaling pathway, leading to the induction of cell cycle-dependent kinase inhibitors, such as p27^Kip1^. On the other hand, a selective defect in signals leading to sphingomyelinase (SMase) activation could confer resistance to apoptosis [9]. It has been demonstrated that the SMase enzyme has different roles on cell fate, depending on its localization; if it resides in the cell membrane or mitochondria [10], it is involved in apoptosis, and if it resides in the cell nucleus [11], it is involved in cell proliferation. In the cell nucleus, SMase regulates sphingomyelin-synthase (SM-synthase) activity; in fact, the reduction of nuclear SM due to the increase of SMase activity stimulates SM synthase [11].

It is known that GM facilitates ROS-mediated sensitization to various anticancer agents in lung cancer cells [1], but if it has an effect on the SM pathway of cancer cells and influences tumor growth have not yet been investigated.

We demonstrated for the first time that GM delays human non-Hodgkin lymphoblastic lymphoma cell growth by stimulating cellular SMase and inhibiting nuclear SMase that, in turn, increases nuclear SM-synthase activity with the enrichment of the nuclear SM pool and overexpression of GAPDH, B2M, CDKN1A and CDKN1B.

## 2. Results

### 2.1. Lymphocyte and SUP-T1 Cell Composition

In lymphocytes, the content of protein was 554 ± 32 µg/10^6^ cells, that of DNA was 86.6 ± 1.77 µg/10^6^ cells, that of RNA was 18.85 ± 3.23 µg/10^6^ cells and that of phospholipids (PLs) was 68.02 ± 3.5 µg/10^6^ cells. The values, expressed as µg/mg of protein, were 156.01 ± 3.20, 33.93 ± 5.82 and 122.44 ± 6.31 for DNA, RNA and PLs, respectively. The SUP-T1 protein content was 588 ± 24 µg/10^6^ cells. No variations were observed for nucleic acid content in cancer cells whereas the total PLs content reduced 1.23 times. The purification level of the nuclear preparation was similar to that previously reported for melanoma cell nuclei [12]. The activity of Glucose-6-Phosphatase present in the nuclei of lymphocyte 1.72% ± 0.40% and that of SUP-T1 was 1.56% ± 0.33% of that present in the whole cells. The NADH-cytochrome-c reductase enzyme activity was 16.20 ± 1.50 nmol/mg protein/min (lymphocytes) and 17.51 ± 1.83 nmol/mg protein/min (SUP-T1) in whole cells, whereas no activity was detected in the nuclei. These data excluded the possibility of contamination by endoplasmic reticulum and mitochondria, as previously reported [12]. DNA, RNA and PL content in nuclei purified from lymphocytes was 494.5 ± 7.62, 89.28 ± 4.91 and 36.51 ± 6.77 µg/mg of protein, respectively. In nuclei purified from SUP-T1 cells, the content of DNA increased 1.14-times, whereas the RNA and lipid content was similar to that of lymphocytes.

### 2.2. What Gentamicin Does in Non-Hodgkin’s T-Cell Human Lymphoblastic Lymphoma Cells

**Figure 1:**
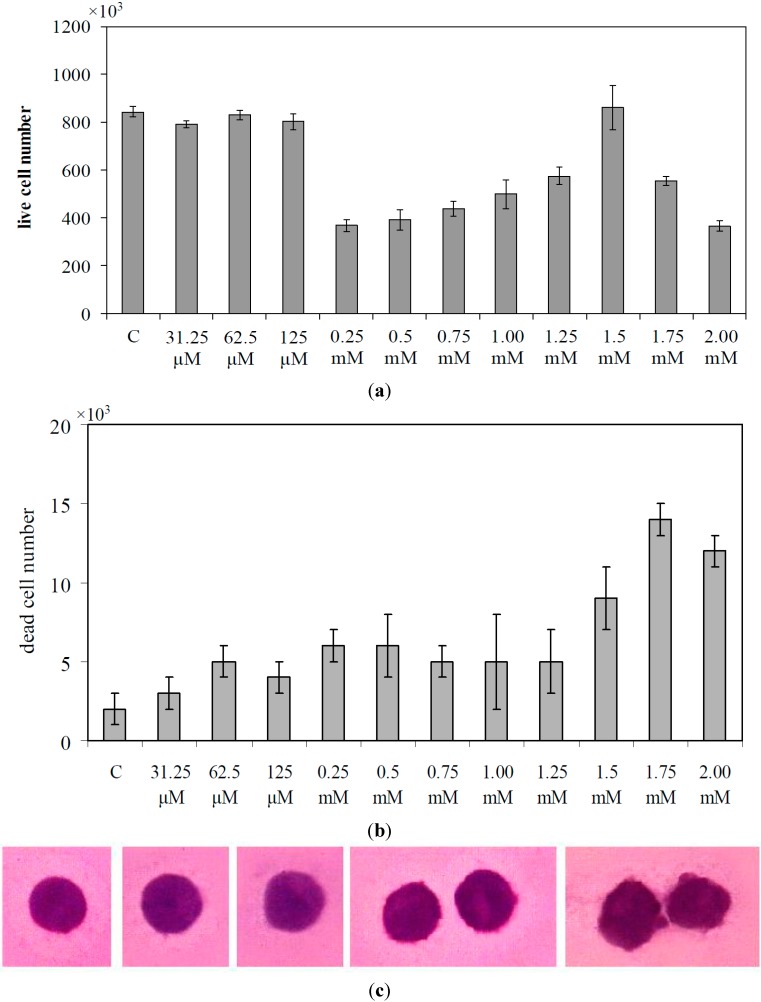
Effect of increasing doses of gentamicin on SUP-T1 cells. (**a**) The number of live cells; (**b**) The number of dead cells; and (**c**) Morphological changes. Data are expressed as the mean ± SD of three independent experiments performed in duplicate. C, control.

**Figure 2:**
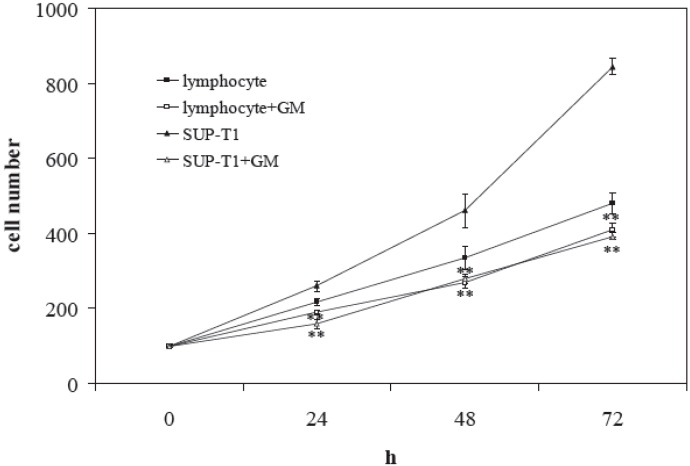
Effect of gentamicin (GM) on lymphocytes and SUP-T1 cells over time. The cell number was evaluated at 0, 24, 48 and 72 h of culture in the presence of 2 mM gentamicin. Data are expressed as the mean ± SD of three independent experiments performed in duplicate. ** *p* < 0.001.

To study the optimal dosage of GM to delay cell growth and to induce cell death, increasing doses of GM from 31.25 μM to 2 mM were used in SUP-T1 cells seeded at a 1 × 10^5^/mL concentration. After 72 h of culture, the control cells were (8.31 ± 0.20) × 10^5^/mL. The results showed that until 125 μM, GM did not reduce cell growth; the 0.25 mM concentration strongly reduced cell growth (Figure 1a), but with low cell death (Figure 1b). Curiously, the inhibitory effect decreased with progressively increasing GM concentration until 1.5 mM. Two millimolar GM was useful to obtain new inhibition of cell proliferation with a value of the cell number similar to that obtained with 0.25 mM, but with the number of cell deaths two-times higher. Hematoxylin-eosin staining of live cells showed that with 2 mM GM, the cells lost their roundness and nuclear protrusions appeared (Figure 1c). Then, 2 mM GM was added to the culture medium of both lymphocytes and SUP-T1 cells seeded at a 1 × 10^5^/m concentration. After 72 h of culture, lymphocytes were (4.8 ± 0.28) × 10^5^/mL and SUP-T1 cells were (8.43 ± 0.22) × 10^5^/mL. The number of lymphocytes decreased 1.14-, 1.24- and 1.17-times, and that of SUP-T1 1.60-, 1.65- and 2.15-times in comparison with the control sample after 24, 48 and 72 h of culture (Figure 2).

**Figure 3:**
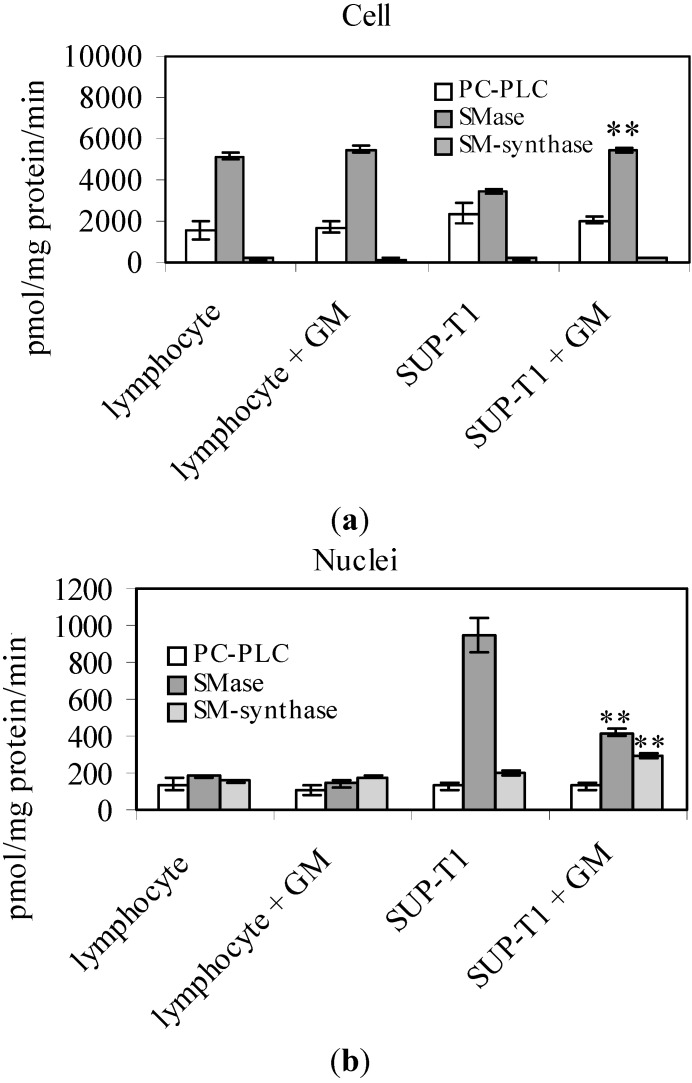

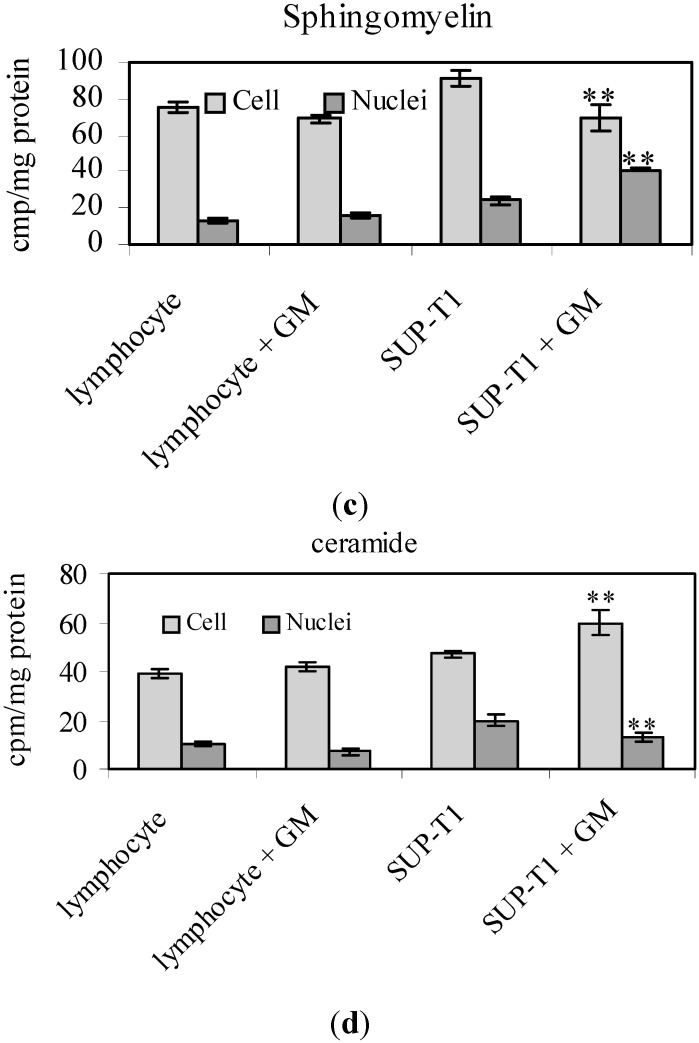
Effect of gentamicin on lipid metabolism in lymphocytes and SUP-T1 cells and in their purified nuclei. The enzyme activity of phosphatidylcholine-dependent phospholipase C (PC-PLC), sphingomyelinase (SMase), sphingomyelin-synthase (SM-synthase) in whole cells (**a**) and in purified nuclei (**b**); ^3^H-palmitic acid incorporation in cells and nuclear sphingomyelin (**c**); ^3^H-palmitic acid incorporation in cells and nuclear ceramide (**d**). Data are expressed as the mean ± SD of three independent experiments performed in duplicate. ** *p* < 0.001.

To investigate the possible mechanism of action, the involvement of SM metabolism was studied after 24 h of culture. In whole cells, 2 mM GM increased SMase activity 1.07-times in lymphocytes and 1.59-times in SUP-T1 cells without variations of SM-synthase activity (Figure 3a). To exclude the possibility that GM acted indiscriminately on several enzymes of lipid degradation, the activity of phosphatidylcholine-dependent phospholipase C (PC-PLC) was studied. No effects were highlighted (Figure 3a). In purified nuclei, the SMase activity of lymphocytes decreased 1.27-times and that of SUP-T1 2.25-times, as well as the SM-synthase activity increased 1.14- and 1.44-times in lymphocytes and SUP-T1 cells, respectively (Figure 3b). No variations were observed for PC-PLC. Incorporation of labelled palmitic acid in SM and ceramide was unchanged significantly in lymphocytes in both whole cells and nuclei (Figure 3c,d). In SUP-T1 cells, the decreased of SM was accompanied by an increase of ceramide, and the opposite happened in purified nuclei (Figure 3c,d).

**Figure 4:**
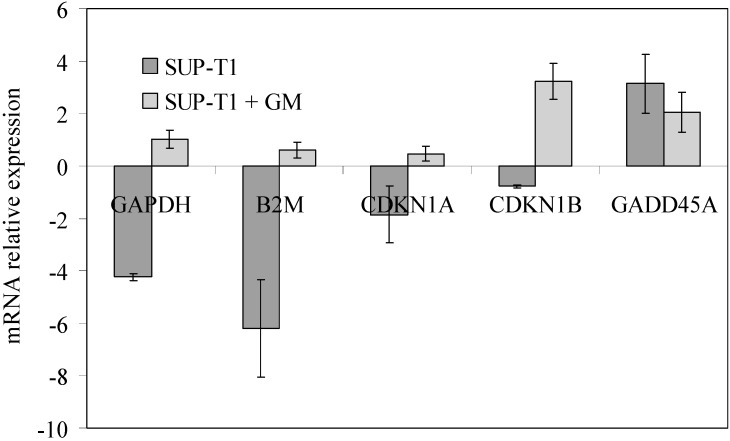
Effect of gentamicin on CDKN1A, CDKN1B, B2M, GAPDH and GADD45A expression. RTqPCR analysis was performed in untreated and gentamicin-treated lymphocytes and SUP-T1 cells. GAPDH has long been used as a default reference gene in quantitative mRNA profiling experiments; however, since its expression varied in cancer, as for many other genes, absolute quantification was preferred, and untreated lymphocytes were used for *Ct* comparison [13]. Data are expressed as the mean ± SD of three independent experiments performed in three PCR replicates.

In association with SM metabolism changes, at 24 h of culture, 2 mM GM changed the gene expression. As shown in Figure 4, where the gene expression refers to that of untreated lymphocytes, in SUP-T1 cells, GAPDH, B2M, CDKN1A and CDKN1B were down-expressed. GM treatment restored the expression of GAPDH, B2M and CDKN1A to values similar to those of lymphocytes (values close to unity) and cause the overexpression of CDKN1B. The specificity of action of GM was supported by the observation that the drug slightly increased the expression of these genes when the treatment was carried out on lymphocytes. On the contrary, GADD45A overexpressed in lymphoma cells and GM did not induce changes (Figure 4). It will be interesting to study in the future the relationship between mRNA and protein content.

## 3. Discussion

Some epidemiological studies have suggested a positive association between antibiotic use and the risk of breast cancer [14]. Rossini *et al.* [15] demonstrated a significantly higher risk of tumor development in metronidazole/ciprofloxacin-treated mice, but not in gentamicin-treated mice compared with controls. We showed for the first time that high doses of GM delayed non-Hodgkin’s T-cell human lymphoblastic lymphoma cell growth and induced cell death. Usually, the daily administration of GM as an antimicrobial therapy is equal to 3 mg/kg/day; if a subject of average weight is used, therefore 62.5 µM GM. Our data indicate that, at this molarity, GM had no effect on lymphoma cells, and to achieve a reduction in cell growth and damage, increasing the molarity by about 30-times in a single administration in required. The reduction of the living cell number without an increase in cell death between 0.25 and 1.5 mM is probably due to the arrest of the cells in the G_1_ phase of the cell cycle, remaining in a quiescent state without suffering damage. We demonstrated that only after 24 h of treatment, 2 mM GM acted on genes involved in cell proliferation. GAPDH, used for long time as a default reference gene in quantitative mRNA profiling experiments, actually varied in response to a range of pathophysiological variables [16]. GAPDH accumulation was correlated with non-apoptotic tumor cell death [17]. Mutation of B2M was implicated in impaired DNA damage response and escape from the immune surveillance mechanisms of T-cell lymphomas [18]. Our data indicated that proliferating lymphoma cells down-expressed GAPDH and B2M, but after GM treatment, useful for inducing cell death, the gene expression increased. We showed a greater speed of growth of lymphoma cells compared to that of the lymphocytes, accompanied by a down-expression of genes for inhibitory proteins of cyclin-dependent kinase (CDK) complexes, such as CDKN1A (p21), which acted at the G_1_/S and G_2_/M cell cycle checkpoints [19], and CDKN1B (p27), which played a critical role in regulating the G_1_/S transition [20]. GM blocked cell growth overexpressing CDKN1A and CDKN1B. In addition, lymphoma cells was characterized by an overexpression, which was not changed by GM, of GADD45A, one of the DNA-damage checkpoint genes that, upon various kinds of stress, maintained genomic integrity in many cell types through DNA repair [21]. Treatment with drugs changes the expression of many genes, and a specific study is needed to identify the housekeeping genes, as previously reported [22]. Since, at this moment, no specific studies have been done on SUP-T1 cells treated with GM to identify the housekeeping genes, we compared mRNA expression of GM-treated cells with the mRNA of control cells, according to Schmittgen and Livak [13]. We have widely demonstrated that nuclear enzymes for lipid metabolism had physicochemical characteristics different from the same enzyme localized in cell and subcellular membranes. Therefore, nuclear lipid metabolism was completely independent of that of other cellular structures and had a specific role on cell life by influencing proliferation, differentiation and/or apoptosis with specific mechanisms [11]. In particular, nuclear SM metabolism regulated cell cycle checkpoints [23]. The demonstration of the existence of an SM cycle inside the nucleus belongs to this last decade of research. SM was degraded by SMase to produce phosphocholine (PPC) and ceramide, and it was restored by SM-synthase by using PPC from PC and free ceramide [11]. During cell proliferation, the activity of nuclear SMase increased during the S phase of the cell cycle, thus favoring the beginning of DNA synthesis [24]. The role of nuclear SMase was opposite that of cellular SMase, which was involved primarily in cell damage [9]. Here, we demonstrated that GM stimulated cellular SMase, by reducing labelled SM and increasing labelled ceramide. In the nucleus, GM inhibited SMase and stimulated SM-synthase by increasing labelled SM. As a consequence, whole cells were enriched in ceramide content and purified nuclei in SM content. The increase of SM content in the nuclei facilitated the formation of nuclear lipid microdomains (NLM), which acted as platforms for gene expression regulation [23]. At the moment, it is not possible to establish a direct relation between the effect of GM on SM metabolism and on gene expression, but we suggest that the increase of nuclear SM might enrich the nucleus of NLM with consequent regulation of gene expression. It is possible that in lymphoblastic lymphoma, high doses of GM induce a beneficial therapeutic outcome.

## 4. Experimental Section

### 4.1. Materials

Non-Hodgkin’s T-cell human lymphoblastic lymphoma (SUP-T1) were from Biological Materials Bank ((ICLC) CBA, Genoa, Italy). Radioactive phosphatidylcholine (PC, l-3-phosphatidyl *N*-methyl-^3^H choline 1,2 dipalmitoyl, 81.0 Ci/mmol), radioactive SM (choline-methyl ^14^C, 54.5 Ci/mol) and ^3^H palmitic acid were obtained from Amersham Pharmacia Biotech (Rainham, Essex, UK). Ecoscint A was obtained from National Diagnostic (Atlanta, GA, USA). PC, SM, non-hydroxy fatty acid ceramide, fetal bovine serum (FBS), RPMI 1640 Medium, PSF (penicillin, streptomycin and fungizone) and GM sulfate salt (477.6 molecular weight) were purchased from Sigma-Aldrich Co. (St. Louis, MO, USA).

### 4.2. Lymphocyte and SUP-T1 Culture

Lymphocytes were extracted from the peripheral blood of three donors (Centro Trasfusionale, Ospedale Silvestrini, Perugia, Italy) by using “Lymphocyte Separation Medium” according to the protocol instructions for use (Lonza Group, Basel, Switzerland). Lymphocytes were cultured in RPMI 1640 medium with penicillin, streptomycin and amphotericin B added in the presence of 10% FCS. SUP-T1 cells were cultured as previously reported [25].

### 4.3. Cell Treatments

To establish the dose-dependent effect of GM on SUP-T1, increasing concentrations of the drug (31.25 μM–2 mM) were added to the culture medium; the cells were counted, and the vitality was determined by trypan blue staining after 72 h, simultaneously with the analysis of cell morphology. For the other experiments, two lots of lymphocytes and SUP-T1 cells were prepared: the control sample (C) without GM and the experimental sample with 2 mM GM. Cell synchronization was performed as previously reported [26], and the cells were examined at 24 h for the enzyme activity assay and gene expression.

### 4.4. Nuclei Purification

The nuclei were isolated and checked for possible cytoplasmic contamination, as previously reported [12].

### 4.5. Biochemical Determinations

Protein content was determined according to Lowry *et al.* [27]. The nucleic acid was extracted and analyzed, and PLs were measured according to Cascianelli *et al.* [28].

### 4.6. Cell Morphology

SUP-T1 cells treated with 0.5, 1, 1.5 and 2 mM GM were fixed, after 72 h of culture, with ethanol 95% for 5 min, submitted to the hematoxylin-eosin (Chroma-Gesellschaft, Stuttgart, Germany) staining method and investigated by using inverted microscopy, EUROMEX FE 2935 (ED, Amhem, The Netherland), equipped with a CMEX 5000 camera system (40× magnification).

### 4.7. Phosphatidylcholine**-**Specific Phospholipase C Assay

The phosphatidylcholine-specific phospholipase C (PC-PLC) activity was detected in whole cells and purified nuclei, as previously reported [29], after 24 h of culture in the absence or presence of 2 mM GM.

### 4.8. Sphingomyelinase Assay

The SMase activity was detected in whole cells and purified nuclei, as previously reported [29], after 24 h of culture, in the absence or presence of 2 mM GM.

### 4.9. Sphingomyelin-Synthase Assay

SM-synthase activity was detected in whole cells and purified nuclei, as previously reported [29], after 24 h of culture, in the absence or presence of 2 mM GM.

### 4.10. [^3^H]-Sphingomyelin and Ceramide Level

The cells from four to six plates were incubated with 1 µCi/mL of [^3^H]-palmitic acid, diluted with cold palmitic acid to a final concentration of 20 nM in culture medium containing 10% FBS for 24 h, as previously reported [30].

### 4.11. Reverse Transcription Quantitative PCR

PCR (RTqPCR) analysis was performed, and the values of gene expression were analyzed as previously reported [22]. Control and experimental lymphocytes and SUP-T1 cells collected after 24 h were used for total RNA extraction performed by using the RNAqueous^®^-4PCR kit (Ambion Inc., Austin, TX, USA). Samples were treated with RNase-free DNase to prevent the amplification of any genomic DNA possibly present. Samples were dissolved in RNase-free water, and the total RNA amount was quantified by measuring the absorbance at 260 nm (A_260_). The purity of RNA was evaluated by using the A_260_/A_280_ ratio. The A_260_/A_230_ ratio also was used as an indicator of the chemical contaminants in nucleic acids. The extracted RNA was immediately frozen and maintained at −80 °C. Before cDNA synthesis, the integrity of RNA was confirmed by 1.2% agarose gel electrophoresis stained with ethidium bromide. cDNA was synthesized using 1 μg total RNA by the High-Capacity cDNA Reverse Transcription kit (Applied Biosystems, Foster City, CA, USA) under the following conditions: 50 °C for 2 min, 95 °C for 10 min, 95 °C for 15 s and 60 °C for 1 min for 40 cycles. RTqPCR was performed using Master Mix TaqMan^®^Gene Expression and the 7.300 RT-PCR instrument (Applied Biosystems), targeting glyceraldehyde-3-phosphate dehydrogenase (GAPDH; Hs 99999905_m1), CDKN1A (Hs 00355782_m1), CDKN1B (Hs 00153277_m1), B2M (Hs 99999907_m1) and GADD45A (Hs 00169255_m1). GAPDH has long been used as a default reference gene in quantitative mRNA profiling experiments; however, since its expression varied in cancer, as for many other genes [15,22], absolute quantification was preferred, and untreated lymphocytes were used for *Ct* comparison [13].

### 4.12. Statistical Analysis

Three experiments performed in duplicate were performed for each analysis. Data are expressed as the mean ± SD, and the *t*-test was used for statistical analysis.

## 5. Conclusions

GM, usually used for the treatment of bacterial infection, delays cancer growth by involving nuclear SM metabolism. It will be interesting in the future to establish if this effect is specific for GM only or if this is a general property of aminoglycosides.
